# Pulmonary Onset of Adult Onset Still's Disease Complicated with Kikuchi Lymphadenitis

**DOI:** 10.1155/2020/8327068

**Published:** 2020-02-19

**Authors:** G. D. E. Kodithuwakku, C. L. Fonseka, S. Nanayakkara, H. M. M. Herath

**Affiliations:** ^1^University Medical Unit, Teaching Hospital Karapitiya, Galle, Sri Lanka; ^2^Department of Medicine, Faculty of Medicine, University of Ruhuna, Galle, Sri Lanka

## Abstract

**Background:**

Adult onset Still's disease (AOSD) is a rare inflammatory disorder with a variety of clinical presentations. Even though pneumonitis and pleurisy are known to occur in AOSD, pulmonary onset presentations are exceedingly rare. *Case Presentation*. We present a 40-year-old male, presenting with fever and bilateral alveolar shadows with pleural effusions mimicking community-acquired severe pneumonia. He was initially treated as severe pneumonia with poor response to broad spectrum antibiotics. Subsequently, he was managed as AOSD-induced pneumonitis, as he fulfilled Yamaguchi criteria. Few weeks later, he developed macular rash and arthralgia with generalized lymphadenopathy with lymph node histology, showing Kikuchi lymphadenitis. He responded well to steroids and had a complete recovery.

**Conclusion:**

Non-infective causes of pneumonitis should be suspected in the setting of poorly resolving pneumonias, especially when microbiological and serological investigations does not support an infective etiology. Presence of systemic symptoms with arthralgia, rash, and disproportionately elevated ferritin level supports the diagnosis of AOSD. Kikuchi lymphadenitis is a reported association with AOSD, and there could be a causal link between the two disorders.

## 1. Introduction

Adult Onset Still's disease (AOSD) is an inflammatory disorder characterized by quotidian fevers, arthritis, and an evanescent rash and considered as a masquerader of many diseases [1]. AOSD can mimic many solid and hematological malignancies, infections (mainly viral), granulomatous diseases, and connective tissue disorders. Therefore, excluding mimicking conditions is pivotal to arrive at diagnosis. Yamaguchi criteria or Fautrel criteria is used as the standard tool for diagnosis, and it included fever, arthralgia, evanescence rash, and leukocytosis as major criteria and sore throat, lymphadenopathy and/or splenomegaly, liver dysfunction, and the absence of rheumatoid factor and antinuclear antibody as minor criteria. Presence of 5 or more criteria was necessary for diagnosis [2,3]. But, these clinical symptoms, signs, or investigation findings may not be apparent during the initial presentation and can be developed progressively hindering early diagnosis.

Parenchymal lung involvement (PLI) is present in less than 5% of AOSD cases and ranges from reticular interstitial opacities to life threatening conditions, such as acute respiratory distress syndrome (ARDS). Moderate-to-severe ARDS frequently occurs early in AOSD with pulmonary involvement usually around 40% of patients having AOSD-related PLI [4]. These patients with ARDS usually respond well to corticosteroids [4]. Pleuritis was mentioned in 10% to 50% in retrospective AOSD series. There are several reported cases of AOSD presenting as pneumonia in the literature, and none of them were complicated with Kikuchi lymphadenitis [4–7]. Considering the non-ARDS PLI in AOSD, the most frequent pattern is chronic interstitial lung disease, and it occurs mainly during systemic AOSD and at any time during the course of the disease. Presence of pericarditis, pleural effusions, and transient pulmonary infiltrates has been observed in 30–40% of patients diagnosed to have AOSD [5]. Pleural effusions are usually small and described as exudative and sterile [8]. Rarely, AOSD may progress to life-threatening adult respiratory distress syndrome, especially if associated with macrophage-activating syndrome [9]. Patients who develop ASOD with pulmonary onset can be misdiagnosed as pneumonia and treated with antibiotics [5].

Lymphadenopathy is a common finding in patients with ASOD [10]. Lymph node biopsies in AOSD have a diverse and dynamic histological spectrum, including atypical paracortical hyperplasia, burnt out histiocytic reaction, exuberant immunoblastic reaction, and follicular hyperplasia [10]. Hence, necrotizing lymphadenitis or granulomatous lymphadenitis is not a recognised feature of AOSD.

Kikuchi–Fujimoto disease (KFD), or histiocytic necrotizing lymphadenitis, is a benign and self-limited disease that mainly affects young women. Patients usually present with localized cervical lymphadenopathy, fever, and leukopenia in up to half of the cases [11]. There are several previously reported co-occurrences of Kikuchi Fujimoto disease and ASOD (Table 1). Both these diseases are rare occurrences, and there may be a causal link for them to occur together. We report a patient with ASOD with pulmonary onset associated with Kikuchi–Fujimoto disease (KFD).

## 2. Case Report

We report a 40-year-old male admitted with episodic high-grade intermittent fever, nonproductive cough, and shortness of breath for a duration of three days. Two years ago, he got admitted to hospital with a similar febrile respiratory illness which was managed as severe pneumonia with a poor response to antibiotics. On examination, the patient was ill-looking, febrile, and dyspneic with a respiratory rate of 28 cycles per minute with bilateral lower zone crepitations in the lungs. He had tachycardia (110 beats per minute) with normal blood pressure. Neurological and abdominal examinations were unremarkable. His oxygen saturation was 90% on air, and arterial blood gas showed a type 1 respiratory failure.

**Figure 1:**
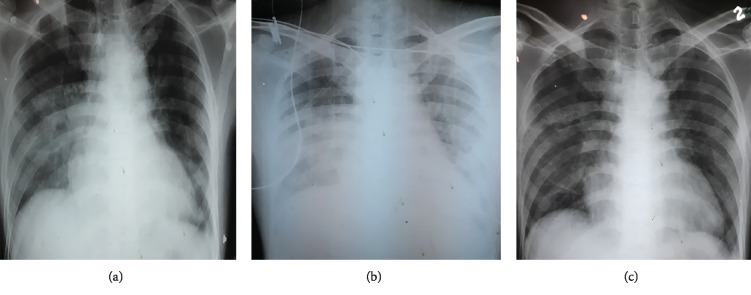
(a) Chest radiograph showing bilateral middle and lower zone alveolar shadows more on the right lung with obliterated left costophrenic angle and (b) chest radiograph revealing more prominent diffuse bilateral opacities and (c) resolution of shadows after steroid treatment.

Initial investigations revealed a white blood cell count of 18,100 per microliter with 90% neutrophils. The platelet count was 22,4000 per microliter. C-reactive protein was 145 milligrams per liter. Chest radiograph revealed bilateral, diffused, middle, and lower zone alveolar shadows (Figure 1(a)). Blood culture and sputum culture for bacteria were negative on 3 repeated occasions. Throat swabs for influenza antigen, myocoplasma serology, and legionella urinary antigen were negative. The patient was initiated on intravenous amoxicillin and clavulanic acid with oral clarithromycin. He did not show a favorable clinical response to treatment, and his clinical symptoms and blood investigations continued to deteriorate despite treatment. On day 5 of admission, he was transferred to an intensive care unit and started on noninvasive ventilation to maintain oxygenation. His antibiotics were changed to intravenous meropenam and vancomycin. Repeated complete blood count had revealed a white blood cell count of 34,400 per microliter; 94% of them were neutrophils, 2% were lymphocytes, and the platelet count was 462,000 per microliter. Other tests showed a C-reactive protein of 433 (<6.0) mg/L, serum total bilirubin of 17 (1.71–20.5) micromoles per liter, serum creatinine was 74 (60–110) micromoles per liter, and blood urea was 19 (7–20) milligrams per deciliter. Serum alanine transaminase of 85 (7–40) u/L, serum aspartate transaminase of 74 (7–40) u/L, and prothrombin time of 14 seconds with an international normalization ratio of 1.3 were shown. Urine analysis was normal.

Serum ferritin was 17,400 (<300) ng/ml. Blood film has shown severe neutrophil leukocytosis with left shift and toxic granules. Ultrasound scan of the chest has revealed consolidations in both middle and lower lobes of the lungs, and there were small bilateral pleural effusions (Figure 1(b)). Ultrasound-guided aspiration of right-side pleural collection revealed 1600 polymorphs per microliter, 50 lymphocytes per microliter, and 250 red cells per microliter. The pleural fluid protein concentration was 31 grams per deciliter, and pleural fluid sugar was 80 (<81) milligrams per deciliter. The pleural fluid lactate dehydrogenase level was 486 (0–248) iu/L, and pH was 7.3. It was an exudate according to light criteria. Pleural fluid bacterial, mycobacterial, and fungal cultures were negative. Repeated blood and sputum cultures were negative. At the same time, he developed continuous central chest pain on day 6 of illness. ECGs revealed dynamic ST segment elevations in leads V2 to V6 and P wave inversion in leads V3 and V4. Cardiac Troponin I titer was 0.58 (0.00–0.40) nanograms per milliliter after 6 hours since the onset of chest pain and 2D echocardiogram was normal. He was managed as a probable myocarditis complicating severe pneumonia.

Over the course of next three weeks, he was given intravenous broad-spectrum antibiotics for 7 days. Due to poor response, antibiotic regimen was changed to intravenous Piperacillin-Tazobactam on the 15^th^ day of illness. It was continued for five more days without much success. The repeat ferritin concentration was found to be > 100,000 ng/ml, which was above the laboratory detection level. To look for non-infective pathology, a comprehensive panel of autoimmune markers was carried out. Serum level of ANA, cANCA, and pANCA, rheumatoid factor, and anti-DsDNA remained normal. Serological tests for HIV and syphilis (VDRL and TPHA) were also negative.

He was started on indomethacin 50 mg three times a day assuming the possibility of Still's disease on the 20^th^ day of illness. Although steroids are the preferred choice in AOSD, possibility of severe pneumonia was not completely ruled out at that time, and starting of steroids was considered dangerous. The patient had a rapid response to initiation of indomethacin and became afebrile within next 24 hours and had significantly improved vital parameters. After 48 hours, the patient was taken off from BiPAP ventilation. He was transferred out of ICU, and he could breathe on normal air on the 23^rd^ day of illness. Piperacillin-tazobactam was continued along with indomethacin for further 12 days. The patient remained afebrile and clinically improved and regained appetite and weight. The white blood cell count came down to 12,100 cells per microliter. C-reactive protein came down to 65 iu/L. Chest radiographs (Figure 1(c)) revealed few bilateral radio-opaque areas in both lower zones of lung fields, indicating resolving pneumonia. He could move and attend his day-to-day routine activities. A complete diagnosis of pulmonary onset AOSD was made, and treatment was continued. Steroids were not considered, as the patient has already responded well.

**Figure 2:**
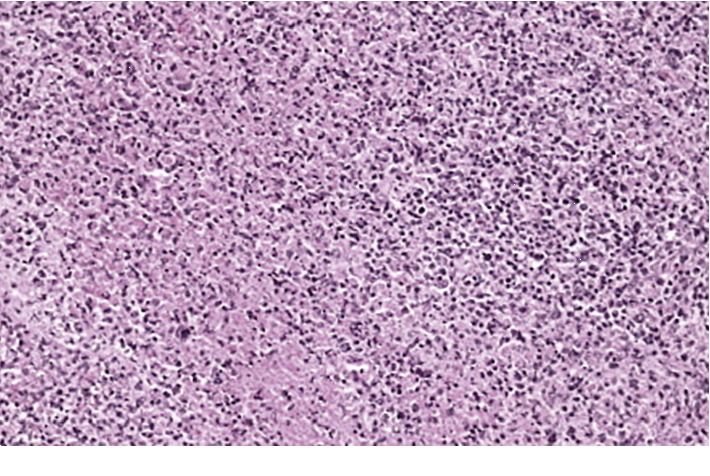
Lymph node histology showing necrotizing lymphadenitis in the paracortical areas of the lymph node.

On the 35^th^ day of illness, the patient developed re-emergence of moderate-grade intermittent fever while on indomethacin. Hospital-acquired infections were excluded by repeating cultures and serological studies. Fever appeared 2 to 3 times each day. He continued to have a good appetite. But, he developed joint pains on both knees and ankles, and he developed a pink-colored maculopapular rash on both upper and lower limbs which lasted for 2 days. He also had generalized lymphadenopathy for the first time during the hospital stay. He also had generalized lymphadenopathy for the first time during the hospital stay which were multiple, firm, mobile, and nontender in bilateral cervical, axillary, and inguinal lymph node groups. His respiratory examination and other system examinations were unremarkable. Left cervical lymph node biopsy was histologically compatible with Kikuchi lymphadenitis that has demonstrated abundant karyorrhectic debris and a proliferation of histiocytes and plasmacytoid cells (Figure 2).

He was given intravenous methyl prednisolone 1 g/daily for three days and later converted to oral prednisolone. All antibiotics were discontinued. Soon after starting steroid treatment, the patient demonstrated signs of complete improvement. He was afebrile, and his joint pains disappeared. Lymph nodes were impalpable after 10 days since the initiation of steroids. The patient was discharged on the 42^nd^ day of illness. On follow-up after first and six months, he continued to have a good health. Oral prednisolone was continued and gradually tailed off over the next 6 months.

## 3. Discussion

This middle-aged gentleman with history of poorly resolving pneumonia two years ago presented with presentation similar to bilateral severe pneumonia and was treated with several combinations of broad-spectrum antibiotics without success. We were unable to isolate an infective organism despite extensive evaluation. Relatively common inflammatory diseases like sarcoidosis, infections such as tuberculosis, and HIV were not detected in screening. He developed very high ferritin 3 weeks later in the disease course and fulfilled Yamaguchi criteria for AOSD. He responded well to initial indomethecin trial, but 10 days after, he developed generalized lymphadenopathy with a macular rash. Histology of lymph node showed necrotizing histiocytic lymphadenitis suggesting Kikuchi–Fujimoto Disease (KFD). His treatment was converted to steroids, and he had a good response with a complete remission of the clinical manifestations.

Adult onset Still's disease has several recognized clinical patterns as monocyclic, recurrent, and chronic. Monocyclic variant occurs once and completely resolves. Chronic form can persist with variable clinical course without disease-free intervals, and the patient will need long-term treatment. Recurrent form occurs in separate cycles, and there are completely disease-free intervals in between [3]. Recurrences are known to occur with worsening intensity as in this case. More notably, his recurrence had a similar spectrum of clinical manifestations to his first presentation.

KFD occurred later in the disease course in this patient, and the clinical features were distinct. Lymph node biopsy plays a crucial role in diagnosis of KFD. AOSD is associated with a wide range of lymph node histological patterns [10]. But, necrotizing inflammation of lymph nodes mimicking KFD has not been reported previously in isolated occurrences of AOSD. It is questionable whether lymph node histology with central necrosis is a finding related to AOSD or Kikuchi disease occurring concurrently with ASOD. Authors hypothesize that this could be an overlap between AOSD and Kikuchi disease which are two rare diseases as the second disease appeared later in the disease course with a distinct set of clinical manifestations. Since both diseases have an autoimmune etiology, there could be similarities in their immunopathogenesis. Also, both illnesses are diagnosed after excluding common etiologies that can lead to similar presentation; hence, this necrotizing lymphadenitis may also be an association of AOSD as KFD occurs in the setting of autoimmune diseases. Kikuchi–Fujimoto disease was hypothesized to be a reactive process. There are indications that there is a defect in the course of apoptotic cell death in both AOSD and KFD [16]. The prognosis of Kikuchi–Fujimoto syndrome is good, and it is a mostly self-limiting disease with a recurrence rate of only about 3%. The course of adult onset Still's disease can be very different [17].

A literature survey revealed several previously reported co-occurrences of AOSD with Kikuchi lymphadenitis (Table 1). Dalugama and Gawarammana. described a case of prolonged pyrexia later diagnosed as KFD who had elevated ferritin levels atypical of AOSD [18]. Another case described a patient suspected to have KFD with a skin rash histology suggesting SLE-like features and then developed features of AOSD after 4 months of initial presentation [19]. These provide more evidence that these two illnesses can be co-occurences in the background of an autoimmune response. KFD is mainly known to cause cervical lymphadenopathy. Most of the previously reported KFD and AOSD co-occurrences demonstrated generalized lymphadenopathy rather than cervical lymphadenopathy. Leukocytosis and markedly elevated ferritin levels would be atypical for KFD alone; therefore, the presence may suggest AOSD [17]. Autoimmune diseases like systemic juvenile idiopathic arthritis, systemic lupus erythematosus (SLE), polymyositis, Sjogren's syndrome, and hemophagocytic lymphohistiocytosis are reported to associate with KFD. A large majority of these cases are associated with SLE compared with other mentioned autoimmune diseases [20].

There had been several co-occurrences of systemic juvenile idiopathic arthritis (JIA) and KFD in the recent literature [16,20,21]. AOSD is considered the adult counter part of JIA, and these reports are also worth noticing due to the close relationship between JIA and AOSD. Ramanan et al. reported a one-year-old girl with JIA and KFD. Onset and clinical manifestation of JIA was followed by lymphadenopathy and lymph node biopsy revealed necrotizing lymphadenitis. She responded to steroids, and lymph nodes also disappeared with steroid treatment. Compared with AOSD, details on patterns of JIA lymph node histology are sparse [16]. Marsili et al. reported an interesting association between Kikuchi disease, macrophage activation syndrome (MAS), and systemic juvenile arthritis with a perforin gene defect in a 15-year-old girl. MAS is known to occur in the setting of rheumatological diseases including JIA and KFD. This is the sole case report with a genetic analysis [21]. Assimakopoulos et al. reported a case of suppurative necrotizing granulomatous lymphadenitis in AOSD in a patient who presented with fever, abdominal pain, episodic skin rash, and mesenteric lymphadenopathy histologically characterized by necrotizing granulomatous adenitis. He responded well to steroids [22]. This co-occurrence of JIA with KFD and the fact that KFD is associated with autoimmune diseases denote that KFD could also be associated with AOSD as well.

Both KFD and AOSD are rare diseases, and co-occurrences of these diseases are unlikely to be a random co-occurence. Autoimmune diseases tend to occur together, but, however, a deeper understanding of KFD occurring together with other inflammatory diseases may shed light on finding clues to the disease etiologies and development of new treatment.

## 4. Conclusion

AOSD can present with acute bilateral pneumonitis leading to respiratory failure mimicking severe community-acquired pneumonia, and the typical disease manifestations of AOSD can occur later in the course of the disease. Lack of response to antibiotic and repeated negative culture results should prompt investigation for a noninfective etiology. Trial of indomethacin or steroids can support the diagnosis but should be carefully decided especially with a suspicion of a septic focus. Kikuchi–Fujimoto disease and adult onset Still's disease may share a common etiology and can coexist.

## Figures and Tables

**Table 1:** Characteristics of reported cases of co-occurrences of AOSD with KFD.

Author/year reported	Age/sex	Clinical features	Investigations	Treatment/recovery	Comment
Cousin et al., 1999 [12]	32 y, male	Spiking fever with axillary, inguinal, and neck lymphadenopathy, arthritis, and cutaneous eruption	Immunohistochemistry of lymph node biopsy CD68 was positive in pathologic paracortical areas. Necrotizing lymphadenitis	Oral prednisolone therapy, 60 mg/day	A relapse was observed a few weeks later during a decrease in steroid dose
Torbio et al., 2014 [13]	22 y, male	Fever, rash, arthritis, and lymphadenopathy	Skin biopsy showed superficial and deep perivascular and periadnexal dermatitis with focal interface changes suggestive of SLE. But, serological investigations were negative for SLE	Anti-IL-1*β* therapy with anakinra	Rapid recovery
Liberatos, 1990 [14]	18 y, male	Fever, rash, arthritis, and lymphadenopathy	Necrotizing lymphadenitis	Steroids	Full recovery
Liberatos, 1990 [14]	47 y, female	Fever, rash, arthritis, and lymphadenopathy	Necrotizing lymphadenitis	NSAIDs, (aspirin)	Full recovery at 3 months follow-up
Ohta et al., 1988 [15]	14 y, female	Fever, rash, arthritis, and lymphadenopathy	Necrotizing lymphadenitis	Steroids	No improvement with NSAIDs treatment
Ohta et al., 1988 [15]	19 y, male	Fever, rash, arthritis, and lymphadenopathy	Necrotizing lymphadenitis	Steroids	Marked improvement
Ohta et al., 1988 [15]	24 y, female	Fever, rash, arthritis, and lymphadenopathy	Necrotizing lymphadenitis		
Index case	40 y, male	Fever and pneumonitis with delayed appearance of rash and lymphadenopathy	Necrotizing lymphadenitis in lymph node histology with abundant karyorrhectic debris and proliferation of histiocytes and plasmacytoid cells. Extremely high serum ferritin >100,000 ng/ml	NSAID (indomethacin) steroids	Initial recovery to NSAIDs and reappearance of symptoms responded to steroids

## References

[1] Still G. F., Garrod A. E. (1897). On a form of chronic joint disease in children. *Journal of the Royal Society of Medicine*.

[2] Bywaters E. G. (1971). Still’s disease in the adult. *Annals of the Rheumatic Diseases*.

[3] Gopalarathinam R., Orlowsky E., Kesavalu R., Yelaminchili S. (2016). Adult onset Still disease: a review on diagnostic workup and treatment options. *Case reports in rheumatology*.

[4] Gerfaud-Valentin M., Cottin V., Jamilloux Y. (2016). Parenchymal lung involvement in adult-onset Still disease. *Medicine*.

[5] Guerrieri A., Angeletti G., Mazzolini M., Bassi I., Nava S. (2017). Pulmonary involvement in adult Still’s disease: case report and brief review of literature. *Respiratory Medicine Case Reports*.

[6] Ibn Yacoub Y., Amine B., Hajjaj-Hassouni N. (2011). A case of adult-onset Still’s disease complicated with atypical pulmonary defect. *Rheumatology International*.

[7] Qi H., Yin C., Xiao H., Duan T. (2014). A rare case of diffuse pulmonary nodules in a patient with adult-onset still’s disease. *Internal Medicine*.

[8] Mahfoudhi M., Gorsane I., Shimi R., Turki S., Abdallah T. B. (2015). Adult onset still’s disease. *International Journal of Clinical Medicine*.

[9] Pouchot J., Sampalis J. S., Beaudet F. (1991). Adult Stillʼs disease. *Medicine*.

[10] Jeon Y. K., Paik H., Park S. (2004). Spectrum of lymph node pathology in adult onset Still’s disease; analysis of 12 patients with one follow up biopsy. *Journal of Clinical Pathology*.

[11] Hutchinson C. (2019). Kikuchi-fujimoto disease archives of pathology & laboratory medicine online. https://www.archivesofpathology.org/doi/full/10.1043/1543-2165-134.2.289.

[12] Cousin F., Grézard P., Roth B., Balme B., Grégoire-Bardel M., Perrot H. (1999). Kikuchi disease associated with Still disease. *International Journal of Dermatology*.

[13] Toribio K. A., Kamino H., Hu S., Pomeranz M., Pillinger M. H. (2015). Co-occurrence of Kikuchi-Fujimoto’s disease and Still’s disease: case report and review of previously reported cases. *Clinical Rheumatology*.

[14] Lyberatos C. (1990). Two more cases of Still disease and Kikuchi’s. *The Journal of Rheumatology*.

[15] Ohta A., Matsumoto Y., Ohta T., Kaneoka H., Yamaguchi M. (1988). Still disease associated with necrotizing lymphadenitis (Kikuchi’s disease): report of 3 cases. *The Journal of Rheumatology*.

[16] Ramanan A. V. (2003). Systemic juvenile idiopathic arthritis, Kikuchi’s disease and haemophagocytic lymphohistiocytosis--is there a link? Case report and literature review. *Rheumatology*.

[17] Sondermann W., Hillen U., Reis A. C., Schimming T., Schilling B. (2015). Kikuchi-fujimoto-syndrom und adulter morbus still. *Der Hautarzt*.

[18] Dalugama C., Gawarammana I. B. (2017). Fever with lymphadenopathy-Kikuchi fujimoto disease, a great masquerader: a case report. *Journal of Medical Case Reports*.

[19] Miura T., Yamamoto T. (2012). Adult-onset Still’s disease presenting lupus erythematosus-like facial erythema associated with Kikuchi’s disease. *European Journal of Dermatology*.

[20] Mahajan T., Merriman R. C., Stone M. J. (2007). Kikuchi-Fujimoto disease (histiocytic necrotizing lymphadenitis): report of a case with other autoimmune manifestations. *Baylor University Medical Center Proceedings*.

[21] Marsili M., Nozzi M., Onofrillo D., Sieni E., Chiarelli F., Breda L. (2015). Kikuchi disease, macrophage activation syndrome, and systemic juvenile arthritis: a new case associated with a mutation in the perforin gene. *Scandinavian Journal of Rheumatology*.

[22] Assimakopoulos S. F., Karamouzos V., Papakonstantinou C., Zolota V., Labropoulou-Karatza C., Gogos C. (2012). Suppurative necrotizing granulomatous lymphadenitis in adult-onset Still disease: a case report. *Journal of Medical Case Reports*.

